# The relationship between sodium concentrations in spot urine and blood pressure increases: a prospective study of Japanese general population: the Circulatory Risk in Communities Study (CIRCS)

**DOI:** 10.1186/s12872-016-0219-1

**Published:** 2016-03-05

**Authors:** Mitsumasa Umesawa, Kazumasa Yamagishi, Hiroyuki Noda, Ai Ikeda, Shinobu Sawachi, Isao Muraki, Choy-Lye Chei, Renzhe Cui, Masanori Nagao, Tetsuya Ohira, Tomoko Sankai, Takeshi Tanigawa, Akihiko Kitamura, Masahiko Kiyama, Hiroyasu Iso, CIRCS Investigators

**Affiliations:** Department of Public Health, Dokkyo Medical University, School of Medicine, 880 Kita-kobayashi, Mibu, Shimotsuga-gun, Tochigi 321-0293 Japan; Department of Public Health Medicine, Faculty of Medicine, University of Tsukuba, 1-1-1 Tennodai, Tsukuba, 305-8575 Japan; Public Health, Department of Social Medicine, Osaka University Graduate School of Medicine, 2-2 Yamadaoka, Suita, 565-0871 Japan; Department of Public Health, Graduate School of Medicine, Juntendo University, 2-1-1 Hongo, Bunkyo-ku, Tokyo, 113-8421 Japan; Biogen Japan Ltd., 14th floor 1-4-1 Nihonbashi, Chuo-ku, Tokyo, 103-0027 Japan; Osaka Center for Cancer and Cardiovascular Diseases Prevention, 1-3-2 Nakamichi, Higashinari-ku, Osaka, 537-0025 Japan; Department of Community Health, Faculty of Medicine, University of Tsukuba, 1-1-1 Tennodai, Tsukuba, 305-8575 Japan

**Keywords:** Sodium, Urine, Blood pressure, Prospective study, Epidemiology

## Abstract

**Background:**

Although several cross-sectional and intervention studies showed that sodium intake or excretion was associated with blood pressure levels, no prospective study has examined the long-term association between sodium excretion in spot urine and blood pressure changes.

**Methods:**

We conducted a prospective study of 889 normotensive subjects (295 men and 594 women, mean age 57.3 years) who underwent the baseline survey including spot urine test in 2005 and the follow-up survey in 2009 to 2011 (mean follow-up period: 5.8 years). We examined the association between sodium concentration in spot urine, a validated index of sodium excretion occurring over 24-h, and blood pressure changes between baseline and follow-up survey in all, non-overweight (body mass index(BMI) ≤ 25 kg/m^2^) and overweight normotensives.

**Results:**

For all subjects, sodium concentrations in spot urine were not associated with either systolic or diastolic blood pressure changes. When stratified by BMI at baseline survey, sodium concentrations were positively associated with systolic blood pressure changes in non-overweight subjects, but not in overweight subjects. After adjustment for age, sex, BMI, alcohol intake status, current smoking and estimated glomerular filtration rate, the multivariable-adjusted mean values of the systolic blood pressure change among non-overweight subjects was +7.3 mmHg in the highest quartiles of sodium concentrations, while it was +3.9 mmHg in the lowest quartile (*P for difference* = 0.021, *P for trend* = 0.040). After further adjustment of baseline blood pressure levels, the association was slightly weakened; the multivariable-adjusted mean values of the systolic blood pressure changes were +7.0 mmHg and +4.2 mmHg (*P for difference* = 0.047, *P for trend* = 0.071).

**Conclusions:**

High sodium concentrations in spot urine were associated with subsequent systolic blood pressure increases among non-overweight normotensive individuals. (272 words)

**Electronic supplementary material:**

The online version of this article (doi:10.1186/s12872-016-0219-1) contains supplementary material, which is available to authorized users.

## Background

The relationship between sodium intake and blood pressure has been investigated since the 1900s and the associations between sodium intake/excretion and blood pressure levels have been studied mainly by using cross-sectional designs [1–6]. Furthermore, a randomized controlled trial of individuals whose systolic blood pressure of 120 to 159 mmHg and/or diastolic blood pressure of 80 to 95 mmHg showed that the reduction in sodium intake from 150 to 50 mmol/day (8.8 to 2.9 g/day salt) lowered both systolic and diastolic blood pressure levels by 4.8 mmHg and 2.6 mmHg, respectively over the 90-day intervention [7]. A clinical experimental study of 14 normotensive men showed that an extreme sodium intake, 800 mmol/day (46.8 g/day salt intake) at three days increased systolic and diastolic blood pressure levels by 8 mmHg and 7 mmHg, respectively [8]. However, no prospective studies have evaluated the long-term effects of high sodium intake or excretion on subsequent blood pressure changes in general populations. For the primary and secondary prevention of hypertension, the investigation of relationship between sodium intake or excretion with subsequent blood pressure changes among general populations is warranted.

**Fig. 1:**
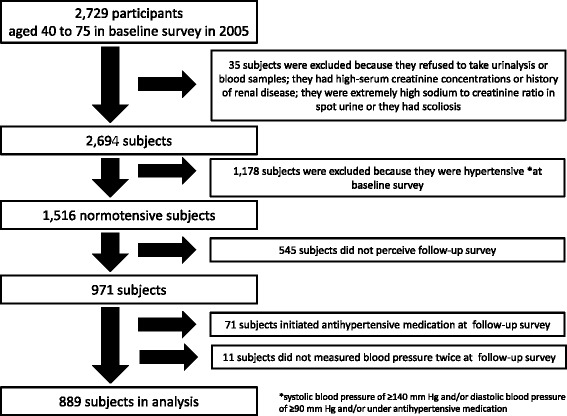
Study subjects

The prevalence of hypertension has been reported to be similar between the Japanese and American populations. According to national surveys, the prevalence of hypertension among 40 to 64-year-old Japanese men and women was 42 % [9], while the prevalence among 45 to 64-year-old US men and women was 41 % [10]. As for subjects aged 65 years or more, the prevalence was 69 % among Japanese and 70 % among Americans. However, the causes or related factors of hypertension may be different in Japanese and Americans. Compared with Americans, Japanese have the lower prevalence of obesity but consume higher amounts of sodium [9, 11, 12]. Mean values of body mass index (BMI) among Japanese adults were found to be 23.4 kg/m^2^ in men and 22.4 kg/m^2^ in women [9], while they were 27.8 kg/m^2^ in US men and 28.1 kg/m^2^ in US women [11]. According to the Japan National Nutrition Survey, mean sodium intake was 4,488 mg/day (11.4 g/day salt) in men and 3,858 mg/day (9.8 g/day salt) in women [9], while in the United States, mean sodium intake was 3,877 mg/day (9.9 g/day salt) in men and 2,896 mg/day (7.4 g/day salt) in women [12]. Therefore, from a public health point of view, it is important to evaluate an effect of sodium on blood pressure levels in Japanese populations, whose mean sodium intake is high.

A “gold standard” to evaluate sodium intake or excretion would be a urine sample collection repeated every 24 h, but it is not easy to conduct such an assay for general populations. Several studies have shown that spot urine is also a practical and useful method to evaluate sodium excretion [13–15]. Using spot urine, we sought to test the hypothesis that urinary sodium excretion is positively associated with subsequent blood pressure increases in a prospective study of a Japanese population.

## Methods

### Population and subjects

The subjects were residents of Kyowa, a rural district of Chikusei City, Ibaraki Prefecture, Japan. The population in the area was 16,778 (8,270 men and 8,508 women), determined by census in 2005. The Kyowa community is a community-based part of the Circulatory Risk in the Community Study (CIRCS) [16], and annual cardiovascular risk surveys have been conducted between mid-November and mid-December since 1981.

There were 2,729 participants (1,037 men and 1,692 women) aged 40 to 75 years old in the 2005 baseline cardiovascular risk survey. We excluded 35 subjects because they refused to take urinalysis (*n =* 8); they refused to take blood samples (*n =* 2); they had high-serum creatinine concentrations (≥ 1.4 mg/dl for men and ≥ 1.2 mg/dl for women) or had a history of renal disease (*n =* 18); extremely high sodium to creatinine ratio in spot urine (≥ 15) (*n =* 4) or non-validated height due to scoliosis (*n =* 3). Of the remaining 2,694 subjects, 1,516 subjects were normotensive and 1,178 subjects were hypertensive (systolic blood pressure of ≥ 140 mm Hg and/or diastolic blood pressure of ≥ 90 mm Hg and/or under antihypertensive medication). Of the normotensive 1,516 subjects, 971 subjects (64.1 %) participated in follow-up cardiovascular risk surveys in 2009 to 2011. We excluded 71 subjects who initiated antihypertensive medication at follow-up surveys. We also excluded 11 subjects because they measured their blood pressure only once (but not twice) at follow-up surveys.

Finally, we used the data from 889 normotensive subjects (295 men and 594 women, mean age 57.3 years) for the analyses (Fig. 1). If the subjects had participated in cardiovascular risk surveys twice or more between 2009 and 2011, we used their most recently acquired data for the analyses: The numbers of subjects we used were 71 in 2009, 91 in 2010 and 727 in 2011.

### Ethics, consent and permissions

This study was approved by the Ethics Committee in University of Tsukuba (12–4, 66–2), Osaka University (13482, 14285) and Dokkyo Medical University (dmu-25004), and institutional review boards of the Osaka Center for Cancer and Cardiovascular Diseases Prevention (26-*rinri*-1). According to the Japanese ethics guidelines, we provided the ethical information on our study at the survey sites and opt out opportunities.

### Population surveys

Individuals who participated in the survey collected their urine in 10-ml plastic urine containers. Concentrations of urine components such as sodium, potassium, urea nitrogen, and creatinine were analyzed using an electrolyte analyzer (Hitachi/Roche; Hitachi, Tokyo, Japan) (Additional file 1). Quality control was undergone three times per day by using normal and abnormal reagent (Consela “Nissui”, Nissui pharmaceutical Co., Tokyo, Japan).

In the baseline survey, arterial systolic blood pressure and the fifth-phase of Korotkoff sounds diastolic blood pressure were measured by well-trained observers using standard mercury sphygmomanometers on the right arm. Observers used large cuff when a subject was large arm circumference. All participants had their blood pressure levels measured twice. In the follow-up survey, participants had their blood pressure levels measured by automated sphygmomanometers (TM-2655P; A&D Company Ltd. Tokyo, Japan) on the right arm twice. According to the validation study of TM-2655 series, the TM-2655 device achieved British Hypertension Society grade A for systolic and A for diastolic blood pressures, and the proportions of values agreeing to within 5 mmHg were 72.5 % for systolic blood pressure and 78.8 % for diastolic blood pressure [17]. In addition, we added 1.0 mmHg for the value of systolic blood pressure and 0.9 mmHg for the value of diastolic blood pressure, because the validation study of TM-2655 device showed that the average difference between mercury sphygmomanometer and TM-2655 reading for systolic blood pressure level was −1.0 ± 5.2 mmHg and that for diastolic blood pressure level was −0.9 ± 4.7 mmHg [17]. The average value of first and second blood pressure measurement were used for the analyses. We calculated blood pressure changes by subtracting blood pressure values in the baseline survey from the values in the follow-up study.

### Validation study

The validity of measurement of the components in spot urine was checked in a subsample of 225 subjects (85 men and 140 women) who underwent 24-h urine collection in 2005 [18]. The concordance rate between the quartiles of urinary sodium concentration in spot urine and of 24-h urinary sodium excretion was 40.0 % for men, 32.1 % for women and 35.1 % for total subjects.

The sodium to creatinine ratio in urine has frequently been used as a marker of sodium excretion during 24-h urine collections [13–15]. In the present study, however, we found a stronger association between sodium concentrations in spot urine and in urine collected over 24-h than between the sodium to creatinine ratio in urine; the Spearman correlation coefficient between sodium concentrations in spot urine and urinary sodium excretion in samples collected over 24-hours was 0.39 (*P*<0.01; sodium excretion (g/day)= 0.032 x sodium concentration (mmol/l) -2.278 (women) + 9.130) , while it was 0.16 between the sodium to creatinine ratio in spot urine, and 24-h urinary sodium excretion. Therefore, we used the sodium concentrations in spot urine as an index of the level of sodium excretion. As for potassium and urea nitrogen, the Spearman correlation coefficients between their concentrations in spot urine and their levels of urinary excretion over 24-h were 0.01 and 0.31, respectively. The Spearman correlation coefficient between the potassium to creatinine ratio in spot urine and 24-h urinary potassium excretions was 0.20. That between of the urea nitrogen to creatinine ratio in spot urine and 24-h urinary urea nitrogen excretions was 0.11.

We calculated estimated glomerular filtration rate (eGFR) (ml/min/1.73 m^2^) according to the following formula, established by the working group of the Japanese Chronic Kidney Disease Initiative: eGFR (ml/min/1.73 m^2^) = 1.94 x (serum creatinine)^-1.094^ x (age)^-0.287^ for men and 1.94 x (serum creatinine)^-1.094^ x (age)^-0.287^ x 0.739 for women [19].

### Statistical analysis

The analysis was performed for blood pressure changes according to quartiles of sodium concentrations in spot urine. We present the combined data for men and women, because we tested sex interaction for the each analysis and found no significant interactions (*P for interaction* = 0.96 for systolic blood pressure and *P for interaction* = 0.36 for diastolic blood pressure). We performed the stratified analysis by the presence or absence of overweight (BMI ≥ 25 kg/m^2^) because sodium sensitivity can be enhanced in the overweight subjects [20, 21].

Age- and sex-adjusted means and proportions of confounding variables were calculated and tested by analysis of covariance. We used the following formula to calculate the HbA1c value based on the JDS value and the National Glycohemoglobin Standardization Program (NGSP) value: HbA1c (NGSP) = 1.02 × HbA1c(JDS) + 0.25 % [22]. We defined diabetes when HbA1c level was ≥ 6.5 % and/or medicated for diabetes mellitus. Age- and sex-adjusted, and multivariable-adjusted mean values of changes in systolic and diastolic blood pressure were also calculated and tested by the analysis of covariance. The mean values of blood pressure changes associated with changes in one standard deviation (1-SD) increment of sodium concentrations were calculated using the multivariate linear regression analysis.

The confounding variables for adjustment included age (years), sex, BMI (kg/m^2^), drinking status (never, ex-drinkers, current drinkers of ethanol at 1 to 22 g/day and ≥ 23 g/day), current smoking (yes or no) and eGFR (ml/min/1.73 m^2^). We also used the baseline value of systolic or diastolic blood pressure level as confounding variable.

We used SAS version 9.3 software (SAS Institute, Cary, NC) for all analyses. *P* values < 0.05 (two-tailed) were considered statistically significant.

## Results

The distribution of sodium concentration in spot urine among all subjects is illustrated in Additional file 1: Figure S1. Sodium concentrations ranged from 19 to 307 mmol/L with the peak at 101 mmol/L. The baseline characteristics according to quartiles of sodium concentrations in spot urine are shown in Table 1. For all subjects and non-overweight subjects, compared with the lowest quartile of sodium concentrations, the highest quartile had higher means of BMI, but not proportions of diabetes mellitus. For non-overweight subjects, compared with the lowest quartile of sodium concentrations, the highest quartile had higher means of eGFR. For overweight subjects, compared with the lowest quartile of sodium concentrations, the highest quartile had lower proportion of current smoker, and the secondary highest quartiles had higher mean diastolic blood pressure.

**Table 1:** Age- and sex-adjusted characteristics according to quartiles of sodium concentrations in spot urine

	Sodium concentration quartiles in spot urine			
	1 (low)	2	3	4 (high)
All subjects (*n =* 889)				
Number	220	225	220	224
Median sodium concentration (mmol/l)	66	107	145	193
Range of sodium concentration (mmol/l)	19–94	82–137	119–176	163–307
Age (years)	57.7	56.9	58.3	56.3
Range of age (years)	40–75	40–75	40–75	40–75
Women (%)	67	67	67	67
Body mass index (kg/m^2^)	22.4 ± 0.2^a^	22.6 ± 0.2	22.9 ± 0.2	23.2 ± 0.2**
Current drinker (%)	27	30	28	33
Current smoker (%)	13	14	13	13
Systolic blood pressure levels (mmHg)	118.4 ± 0.8	117.8 ± 0.8	118.7 ± 0.8	118.2 ± 0.8
Diastolic blood pressure levels (mmHg)	72.7 ± 0.5	72.4 ± 0.5	74.0 ± 0.5	73.0 ± 0.5
Diabetes mellitus (%)	8	6	3**	5
eGFR(ml/min/1.73 m^2^)	83.2 ± 1.1	83.7 ± 1.1	83.2 ± 1.1	85.5 ± 1.1
Non-overweight subjects (BMI < 25) (*n =* 700)				
Number	175	174	176	175
Median sodium concentration (mmol/l)	65	104	144	190
Range of sodium concentration (mmol/l)	19–91	81–133	118–169	161–304
Age (years)	57.6	56.7	58.0	56.3
Range of age (years)	40–75	40–75	40–75	40–75
Women (%)	68	68	68	68
Body mass index (kg/m^2^)	21.5 ± 0.1	21.7 ± 0.1	21.7 ± 0.1	22.1 ± 0.1**
Current drinker (%)	25	31	32	32
Current smoker (%)	13	14	11	15
Systolic blood pressure levels (mmHg)	117.8 ± 0.9	117.0 ± 0.9	117.6 ± 0.9	117.0 ± 0.9
Diastolic blood pressure levels (mmHg)	72.3 ± 0.6	72.2 ± 0.6	73.1 ± 0.6	72.1 ± 0.6
Diabetes mellitus (%)	7	5	4	4
eGFR(ml/min/1.73 m^2^)	82.9 ± 1.2	84.7 ± 1.2	84.2 ± 1.2	86.3 ± 1.2*
Overweight subjects (BMI ≥ 25) (*n =* 189)				
Number	46	48	48	47
Median sodium concentration (mmol/l)	71	117	154	203
Range of sodium concentration (mmol/l)	20–112	90–149	127–185	170–307
Age (years)	57.6	59.6	58.2	55.8
Range of age (years)	40–70	41–73	43–74	40–74
Women (%)	63	63	60	64
Body mass index (kg/m2)	26.9 ± 0.2	26.5 ± 0.2	26.5 ± 0.2	26.9 ± 0.2
Current drinker (%)	28	20	26	38
Current smoker (%)	20	9	18	4*
Systolic blood pressure levels (mmHg)	120.5 ± 1.4	121.3 ± 1.4	122.8 ± 1.4	122.3 ± 1.4
Diastolic blood pressure levels (mmHg)	72.8 ± 1.1	75.6 ± 1.1	77.1 ± 1.1**	75.3 ± 1.1
Diabetes mellitus (%)	15	4*	1**	10
eGFR(ml/min/1.73 m^2^)	83.8 ± 2.5	79.2 ± 2.4	80.2 ± 2.4	83.1 ± 2.5

Difference from the lowest quartile: *P < 0.05, **P < 0.01

^a^Mean ± SE (all such values)

Mean values in systolic and diastolic blood pressures at baseline and follow-up surveys among all subjects are illustrated in Additional file 2: Figure S2. Mean values of blood pressure increased by 5.1 mmHg for systolic and by 1.0 mmHg for diastolic during the follow-up. The change in diastolic blood pressure was larger in overweight than in non-overweight subjects, and the change in systolic blood pressure was larger in ages of 60-74 than in ages of 40-59. There was no sex difference in the changes if systolic and diastolic blood pressures (Additional file 3: Figure S3). Table 2 shows age- and sex-adjusted, and multivariable-adjusted blood pressure changes according to quartiles and 1-SD (53 to 55 mmol/l) increment of sodium concentrations in spot urine among all, non-overweight and overweight subjects. For all subjects, after adjustment for age, sex, BMI, drinking status, current smoking and eGFR, the mean value of systolic blood pressure changes associated with 1-SD increment of sodium concentrations was +0.9 mmHg (*P* = 0.060). The corresponding mean value was +6.8 mmHg in the highest quartile of sodium concentrations while it was +4.5 mmHg in the lowest quartile (*P* for difference = 0.078). For non-overweight subjects, the multivariable-adjusted mean of systolic blood pressure changes associated with 1-SD increment of sodium concentrations was +1.1 mmHg (*P* = 0.040). The corresponding mean value was +7.3 mmHg in the highest quartile of sodium concentrations, while it was +3.9 mmHg in the lowest quartile (*P* for difference = 0.021). After further adjustment for baseline blood pressure levels, these association were weakened slightly; the multivariable-adjusted mean of systolic blood pressure changes associated with 1-SD increment of sodium concentrations was +0.9 mmHg (*P* = 0.071). The corresponding mean value was +7.0 mmHg in the highest quartile of sodium concentrations, while it was +4.2 mmHg in the lowest quartile (*P* for difference = 0.047). For overweight subjects, no significant associations were observed between sodium concentrations and systolic blood pressure changes. For all categories, no significant changes were observed for diastolic blood pressure. We tested the interactions of sodium concentrations with body mass index, and found no significant interaction between them.

**Table 2:** Multivariable-adjusted blood pressure changes in normotensives according to quartiles and 1-SD increment of sodium concentrations

	Sodium concentration quartiles in spot urine				
	1(low)	2	3	4(high)	1-SD^a^
All subjects (*n =* 889)					
Number	220	225	220	224	54 mmol/l
Changes in systolic blood pressure level (mmHg)					
Age- and sex-adjusted	4.3	4.1	5.2	6.9	1.0*
Multivariable-adjusted^b^	4.5	4.2	5.1	6.8	0.9
Multivariable-adjusted^c^	4.6	4.1	5.2	6.6	0.8
Changes in diastolic blood pressure level (mmHg)					
Age- and sex-adjusted	0.9	0.6	0.3	2.1	0.4
Multivariable-adjusted^b^	1.0	0.7	0.3	2.1	0.4
Multivariable-adjusted^d^	0.9	0.4	0.7	1.9	0.4
Non-overweight subjects (BMI < 25) (*n =* 700)					
Number	175	174	176	175	53 mmol/l
Changes in systolic blood pressure level (mmHg)					
Age- and sex-adjusted	3.8	4.2	4.2	7.3*	1.1*
Multivariable-adjusted^b^	3.9	4.3	4.1	7.3*	1.1*
Multivariable-adjusted^c^	4.2	4.1	4.2	7.0*	0.9
Changes in diastolic blood pressure level (mmHg)					
Age- and sex-adjusted	0.8	0.5	0.1	2.3	0.4
Multivariable-adjusted^b^	0.8	0.5	0.0	2.3	0.4
Multivariable-adjusted^d^	0.8	0.4	0.3	2.0	0.3
Overweight subjects (BMI ≥ 25) (*n =* 189)					
Number	46	48	48	47	55 mmol/l
Changes in systolic blood pressure level (mmHg)					
Age- and sex-adjusted	5.9	5.0	7.1	6.3	0.2
Multivariable-adjusted^b^	5.6	5.0	7.0	6.7	0.4
Multivariable-adjusted^c^	5.3	5.0	7.3	6.8	0.6
Changes in diastolic blood pressure level (mmHg)					
Age- and sex-adjusted	2.5	−0.3	0.5	2.9	0.6
Multivariable-adjusted^b^	2.4	−0.4	0.6	3.0	0.7
Multivariable-adjusted^d^	1.4	−0.1	1.4	2.9	0.9

Difference from the lowest quartile: *P < 0.05

^a^1-SD corresponed to 54 mol/L for all subjects, 53 mol/L for non-overweight subjects and 55 mmol/L for overweight subjects

^b^Adjusted for age (years), sex, body mass index (kg/m^2^), drinking status (never, ex-drinkers, current drinkers of ethanol at 1 to 22 g/day and ≥23 g/day), current smoking (yes or no) and baseline eGFR value (ml/min/1.73 m^2^)

^c^Adjustment further for baseline systolic blood pressure levels (mmHg)

^d^Adjustment further for baseline diastolic blood pressure levels (mmHg)

Additionally, we examined the association between sodium/creatinine ratio and blood pressure changes; however, the association was not statistically significant. The multivariable-adjusted means of systolic and diastolic blood pressure changes associated with 1-SD increment of sodium/creatinine ratio were −0.6 mmHg (*P* = 0.187) and +0.1 mmHg (*P* = 0.728) for all subjects, respectively. We also examined the association between sodium/potassium ratio and blood pressure changes; however, the association was null. The multivariable-adjusted means of systolic and diastolic blood pressure changes associated with 1-SD increment of sodium/potassium ratio were −0.3 mmHg (*P* = 0.515) and 0.0 mmHg (*P* = 0.990) for all subjects, respectively. Those associations did not differ materially between non-overweight and overweight subjects.

## Discussion

The main finding of this study was that sodium concentrations in spot urine were positively associated with subsequent systolic blood pressure changes in non-overweight normotensives.

A cross-sectional study in the United Kingdom, consisting of 23,104 men and women aged 45 to 79 years, showed positive associations between the ratio of urinary sodium to creatinine in spot urine samples and systolic and diastolic blood pressure levels in both men and women [2]. A cross-sectional study of 1,120 Japanese men and women aged 30-years or over showed a positive association between the ratio of urinary sodium to creatinine, and systolic and diastolic blood pressure levels in women, but not in men [3]. A cross-sectional study of 7,441 Japanese women aged 40- to 69-years showed a positive association between estimated 24-h sodium excretion and systolic and diastolic blood pressure level [4]. In the present study, positive associations between sodium concentrations in spot urine and subsequent increases in systolic blood pressure levels were observed for men and women without sex interaction.

Sodium concentrations in spot urine were significantly associated with systolic blood pressure changes and but not with diastolic blood pressure changes in the present study. According to a previous interventional study, the reduction of dietary sodium intake (−44 mmol/day) was significantly associated with both systolic and diastolic blood pressure levels (−1.7 mmHg and −0.9 mmHg, respectively). The association was stronger in systolic blood pressure levels than in diastolic blood pressure levels [23].

Several mechanisms may be involved in the association between sodium excretion and increments in blood pressure levels. First, human kidneys cannot excrete excess sodium completely and thus accumulate water to decrease sodium concentrations in extracellular fluid [24]. This leads to an increase in extracellular fluid and raises pressure in vessels. Second, the excess sodium in extracellular fluid affects the physiology of vascular smooth muscle cells [24]. Increased extracellular sodium inhibits sodium-potassium pumps in arterial vascular smooth muscle cells and raises intracellular sodium concentrations. Excessive intracellular sodium concentrations stimulate sodium-calcium pumps and raise intracellular calcium concentrations, which stimulate actin-myosin interaction leading to vascular contraction. Third, the renal excretion of excess sodium in the circulation needed blood pressure increment as the Guyton auto-regulation theory [25]. Fourth, excess sodium intake may damage the renal function, leading to a blood pressure increase. In the present study, for total subjects, the multivariable-adjusted mean value of eGFR in the follow-up survey was significantly lower in the highest quartile of sodium concentrations in urine, compared with the lowest. The multivariable-adjusted mean eGFR was 74.9 ml/min/1.73 m^2^ in the highest quartile, while it was 76.7 ml/min/1.73 m^2^ in the lowest quartile (*P* for difference = 0.038).

In the present study, we found the significant association between sodium concentrations in spot urine and blood pressure changes in non-overweight but not overweight normotensives. The finding did not corroborate an implication that overweight or metabolic syndrome may enhance salt sensitivity [20, 21], making the salt-blood pressure association stronger. It is possible that overweight may mask that association because of its strong effect on blood pressure.

The strength of the present study is that we conducted a prospective study of a community-based Japanese population sample. Prospective studies can show certain causal relationships and more relevant results compared with cross-sectional studies.

The limitations of the present study warrant discussion. First, the correlation between sodium concentrations in spot urine and urine samples collected was very moderate. However, any errors concerning the misclassification were nondifferential and would have attenuated the association between sodium concentration and blood pressure changes. Second, we changed the device of blood pressure measurement from strandard mercury to automated sphygmomanometers in 2009 (the beginning of the follow-up study). However, the validation study allowed us to adjust the values of blood pressure between the two devices. Third, the follow-up rate of the present study was 64 %. However, there was no significant difference in baseline survey between subjects who participated in follow-up surveys and who did not, except for the proportion of current smoker. Subjects who participated in follow-up surveys showed the lower proportion of current smoker at baseline compared with subjects who did not (15 % vs. 21 %, *P <* 0.001). Because current smoking was not associated with blood pressure changes, such a selection bias unlikely affect our results. Fourth, we could not evaluate the changes of sodium intake between baseline and follow-up surveys because we did not measure sodium concentrations in spot urine repeatedly. Fifth, we have no information about education status, income and other social factors as potential confounding factors.

## Conclusion

High sodium concentrations in spot urine, an index of sodium excretion, was positively associated with future systolic blood pressure changes among non-overweight normotensive Japanese.

## Additional files

Additional file 1: Figure S1. Distribution of sodium concentrations in spot urine among all subjects. (PPTX 85.3 kb) Additional file 2: Figure S2. Mean values of systolic and diastolic blood pressures at baseline and follow-up surveys among all subjects. (PPTX 82 kb) Additional file 3: Figure S3. The changes in systolic and diastolic blood pressures, stratified by overweight, age and sex. (PPTX 99.8 kb)

## Abbreviations

BMI: body mass index
CIRCS: circulatory risk in the community study
1-SD: one standard deviation

## References

[1] Intersalt Cooperative Research Group (1988). INTERSALT: an international study of electrolyte excretion and blood pressure: results for 24 h urinary sodium and potassium excretion. BMJ.

[2] Khaw KT, Bingham S, Welch A, Luben R, O’Brien E, Wareham N (2004). Blood pressure and urinary sodium in men and women: the Norfolk Cohort of the European Prospective Investigation into Cancer (EPIC-Norfolk). Am J Clin Nutr.

[3] Kihara M, Fujikawa J, Ohtaka M, Mano M, Nara Y, Horie R (1984). Interrelationships between blood pressure, sodium, potassium, serum cholesterol, and protein intake in Japanese. Hypertension.

[4] Takemori K, Mikami S, Nihira S, Sasaki N (1989). Relationship of blood pressure to sodium and potassium excretion in Japanese women. Tohoku J Exp Med.

[5] Yamori Y, Liu L, Ikeda K, Mizushima S, Nara Y, Simpson FO (2001). WHO Cardiovascular Diseases and Alimentary Comparison (WHO-CARDIAC) Study. Different associations of blood pressure with 24-hour urinary sodium excretion among pre- and post-menopausal women. WHO Cardiovascular Diseases and Alimentary Comparison (WHO-CARDIAC) Study. J Hypertens.

[6] Zhao L, Stamler J, Yan LL, Zhou B, Wu Y, Liu K (2004). Blood pressure differences between northern and southern Chinese: role of dietary factors: the International Study on Macronutrients and Blood Pressure. Hypertension.

[7] Sacks FM, Svetkey LP, Vollmer WM, Appel LJ, Bray GA, Harsha D (2001). Effects on blood pressure of reduced dietary sodium and the Dietary Approaches to Stop Hypertension (DASH) diet. N Engl J Med.

[8] Luft FC, Rankin LI, Bloch R, Weyman AE, Willis LR, Murray RH (1979). Cardiovascular and humoral responses to extremes of sodium intake in normal black and white men. Circulation.

[9] Ministry of Health, Labour and Welfare (2011). Kokumin Kennko-Eiyo no Gennjyo (The National Health and Nutrition Survey in Japan, 2008).

[10] Keenan NL, Rosendorf KA (2011). Centers for Disease Control and Prevention (CDC). Prevalence of hypertension and controlled hypertension - United States, 2005–2008. MMWR Surveill Summ.

[11] Ogden CL, Fryar CD, Carroll MD, Flegal KM (2004). Mean body weight, height, and body mass index, United States 1960–2002. Adv Data.

[12] Wright JD, Wang CY, Kennedy-Stephenson J, Ervin RB (2003). Dietary intake of ten key nutrients for public health, United States: 1999–2000. Adv Data.

[13] Kawasaki T, Itoh K, Uezono K, Sasaki H (1993). A simple method for estimating 24 h urinary sodium and potassium excretion from second morning voiding urine specimen in adults. Clin Exp Pharmacol Physiol.

[14] Mann SJ, Gerber LM (2010). Estimation of 24-hour sodium excretion from spot urine samples. J Clin Hypertens.

[15] Tanaka T, Okamura T, Miura K, Kadowaki T, Ueshima H, Nakagawa H (2002). A simple method to estimate populational 24-h urinary sodium and potassium excretion using a casual urine specimen. J Hum Hypertens.

[16] Imano H, Kitamura A, Sato S, Kiyama M, Ohira T, Yamagishi K (2009). Trends for blood pressure and its contribution to stroke incidence in the middle-aged Japanese population: the Circulatory Risk in Communities Study (CIRCS). Stroke.

[17] Kobalava ZD, Kotovskaya YV, Babaeva LA, Moiseev VS (2006). Validation of TM-2655 oscillometric device for blood pressure measurement. Blood Press Monit.

[18] Umesawa M, Yamagishi K, Sawachi S, Ikeda A, Noda H, Ikehara S (2010). Urea nitrogen concentrations in spot urine, estimated protein intake and blood pressure levels in a Japanese general population. Am J Hypertens.

[19] Matsuo S, Imai E, Horio M, Yasuda Y, Tomita K, Nitta K (2009). Revised equations for estimated GFR from serum creatinine in Japan. Am J Kidney Dis.

[20] Uzu T, Kimura G, Yamauchi A, Kanasaki M, Isshiki K, Araki S (2006). Enhanced sodium sensitivity and disturbed circadian rhythm of blood pressure in essential hypertension. J Hypertens.

[21] Chen J, Gu D, Huang J, Rao DC, Jaquish CE, Hixson JE (2009). Metabolic syndrome and salt sensitivity of blood pressure in non-diabetic people in China: a dietary intervention study. Lancet.

[22] Kashiwagi A, Kasuga M, Araki E, Oka Y, Hanafusa T, Ito H (2012). International clinical harmonization of glycated hemoglobin in Japan: From Japan Diabetes Society to National Glycohemoglobin Standardization Program values. J Diabetes Investig.

[23] The Trials of Hypertension Prevention Collaborative Research Group (1992). The effects of nonpharmacologic interventions on blood pressure of persons with high normal levels. Results of the Trials of Hypertension Prevention, Phase I. JAMA.

[24] Adrogué HJ, Madias NE (2007). Sodium and potassium in the pathogenesis of hypertension. N Engl J Med.

[25] Guyton AC, Coleman TG, Cowley AV, Scheel KW, Manning RD, Norman RA (1972). Arterial pressure regulation. Overriding dominance of the kidneys in long-term regulation and in hypertension. Am J Med.

