# Erratum to: The relationship between sodium concentrations in spot urine and blood pressure increases: a prospective study of Japanese general population: the Circulatory Risk in Communities Study (CIRCS)

**DOI:** 10.1186/s12872-016-0317-0

**Published:** 2016-06-15

**Authors:** Mitsumasa Umesawa, Kazumasa Yamagishi, Hiroyuki Noda, Ai Ikeda, Shinobu Sawachi, Isao Muraki, Choy-Lye Chei, Renzhe Cui, Masanori Nagao, Tetsuya Ohira, Tomoko Sankai, Takeshi Tanigawa, Akihiko Kitamura, Masahiko Kiyama, Hiroyasu Iso, CIRCS Investigators

**Affiliations:** Department of Public Health, Dokkyo Medical University, School of Medicine, 880 Kita-kobayashi, Mibu, Shimotsuga-gun, Tochigi 321-0293 Japan; Department of Public Health Medicine, Faculty of Medicine, University of Tsukuba, 1-1-1 Tennodai, Tsukuba, 305-8575 Japan; Public Health, Department of Social Medicine, Osaka University Graduate School of Medicine, 2-2 Yamadaoka, Suita, 565-0871 Japan; Department of Public Health, Graduate School of Medicine, Juntendo University, 2-1-1 Hongo, Bunkyo-ku, Tokyo, 113-8421 Japan; Biogen Japan Ltd., 14th floor 1-4-1 Nihonbashi, Chuo-ku, Tokyo, 103-0027 Japan; Osaka Center for Cancer and Cardiovascular Diseases Prevention, 1-3-2 Nakamichi, Higashinari-ku, Osaka, 537-0025 Japan; Department of Community Health, Faculty of Medicine, University of Tsukuba, 1-1-1 Tennodai, Tsukuba, 305-8575 Japan

## Erratum

Unfortunately, the original version of this article [1] contained an error.

We would like to correct a formula in “Validation study” section in page 3.

We wrote that “sodium excretion (g/day) = 0.032 x sodium concentration (mmol/l) -2.278 (women) + 9.130”, however, we would like to replace this with “NaCl excretion (g/day) = 0.032 x sodium concentration (mmol/l) -2.278 (women) + 9.130”.
