# Supplementary figures and images for: The relationship between sodium concentrations in spot urine and blood pressure increases: a prospective study of Japanese general population: the Circulatory Risk in Communities Study (CIRCS)
Source: BMC Cardiovasc Disord. 2016 Mar 5;16:55. doi: 10.1186/s12872-016-0219-1 (PMC4779198; doi:10.1186/s12872-016-0219-1)

## Slide 1
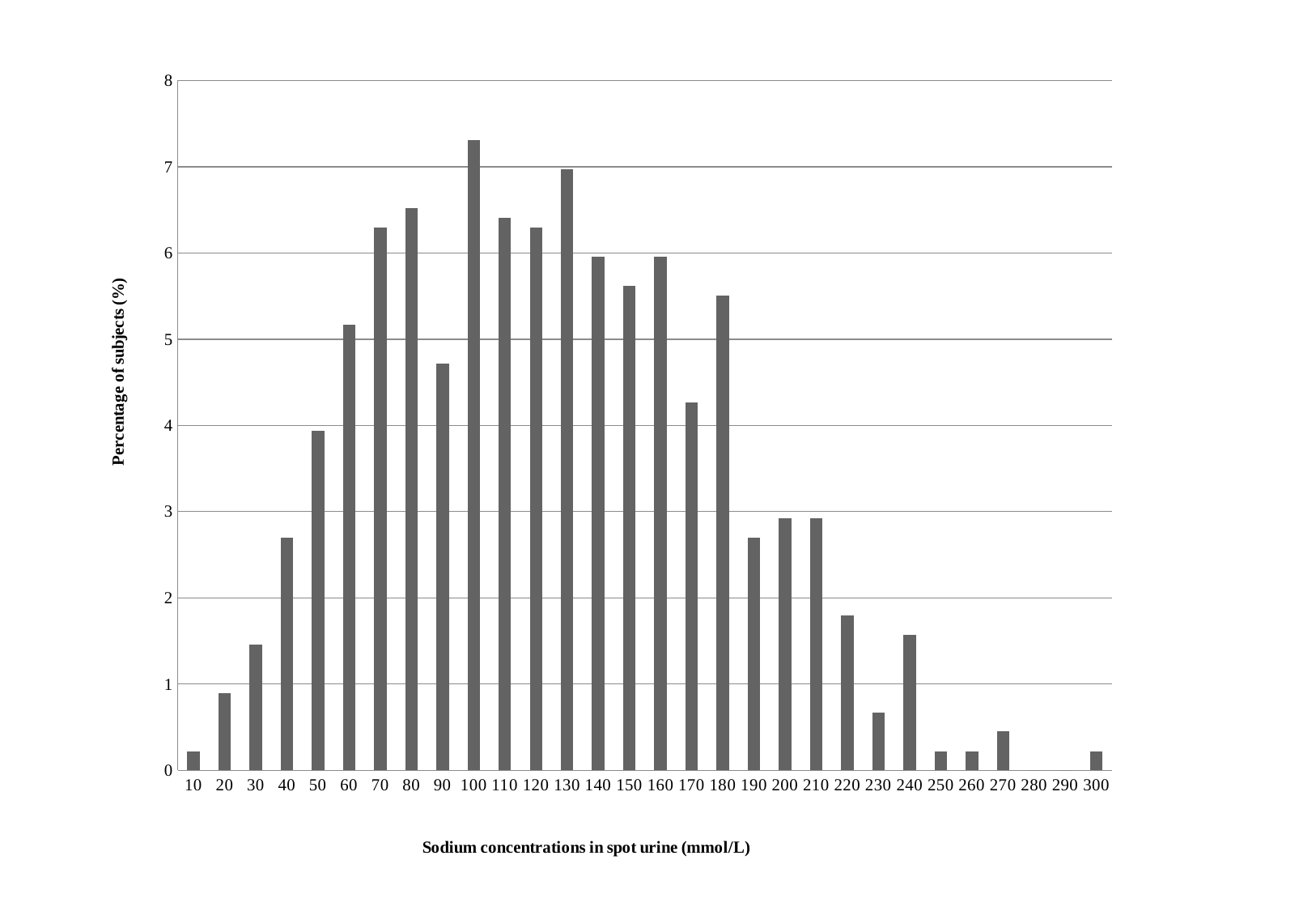

### Chart
| Category | All subjects (n=889) |
|---|---|
| 10 | 0.22 |
| 20 | 0.9 |
| 30 | 1.46 |
| 40 | 2.7 |
| 50 | 3.94 |
| 60 | 5.17 |
| 70 | 6.3 |
| 80 | 6.52 |
| 90 | 4.72 |
| 100 | 7.31 |
| 110 | 6.41 |
| 120 | 6.3 |
| 130 | 6.97 |
| 140 | 5.96 |
| 150 | 5.62 |
| 160 | 5.96 |
| 170 | 4.27 |
| 180 | 5.51 |
| 190 | 2.7 |
| 200 | 2.92 |
| 210 | 2.92 |
| 220 | 1.8 |
| 230 | 0.67 |
| 240 | 1.57 |
| 250 | 0.22 |
| 260 | 0.22 |
| 270 | 0.45 |
| 280 | 0.0 |
| 290 | 0.0 |
| 300 | 0.22 |

Supplement: Additional file 1: Figure S1. — Distribution of sodium concentrations in spot urine among all subjects. (PPTX 85.3 kb) [file 12872_2016_219_MOESM1_ESM.pptx]

## Slide 1
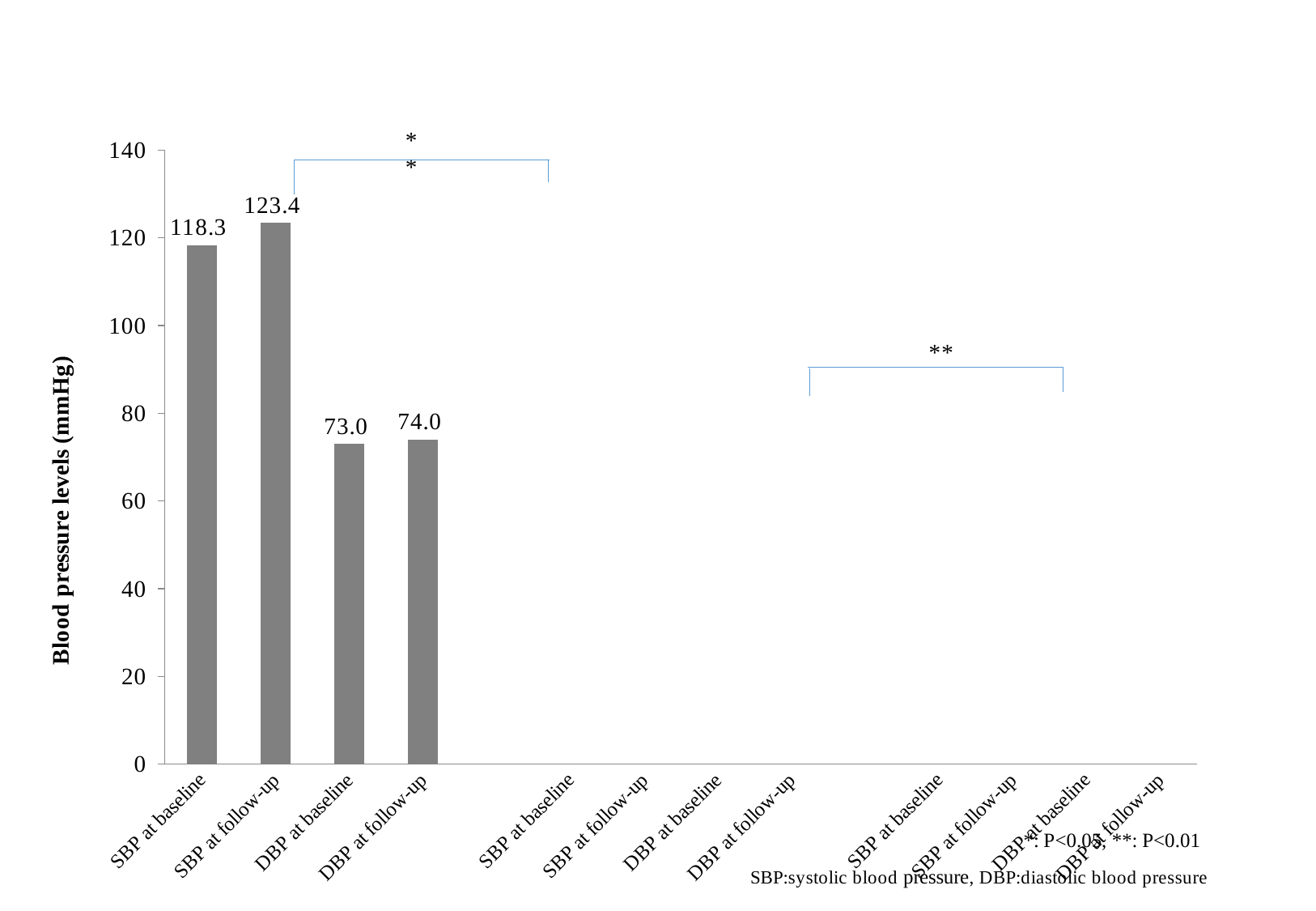

[unsupported chart]

Supplement: Additional file 2: Figure S2. — Mean values of systolic and diastolic blood pressures at baseline and follow-up surveys among all subjects. (PPTX 82 kb) [file 12872_2016_219_MOESM2_ESM.pptx]
