# Supplementary material for: The relationship between sodium concentrations in spot urine and blood pressure increases: a prospective study of Japanese general population: the Circulatory Risk in Communities Study (CIRCS)
Source: BMC Cardiovasc Disord. 2016 Mar 5;16:55. doi: 10.1186/s12872-016-0219-1 (PMC4779198; doi:10.1186/s12872-016-0219-1)
Supplement: Additional file 3: Figure S3. — The changes in systolic and diastolic blood pressures, stratified by overweight, age and sex. (PPTX 99.8 kb) [file 12872_2016_219_MOESM3_ESM.pptx]

## Slide 1
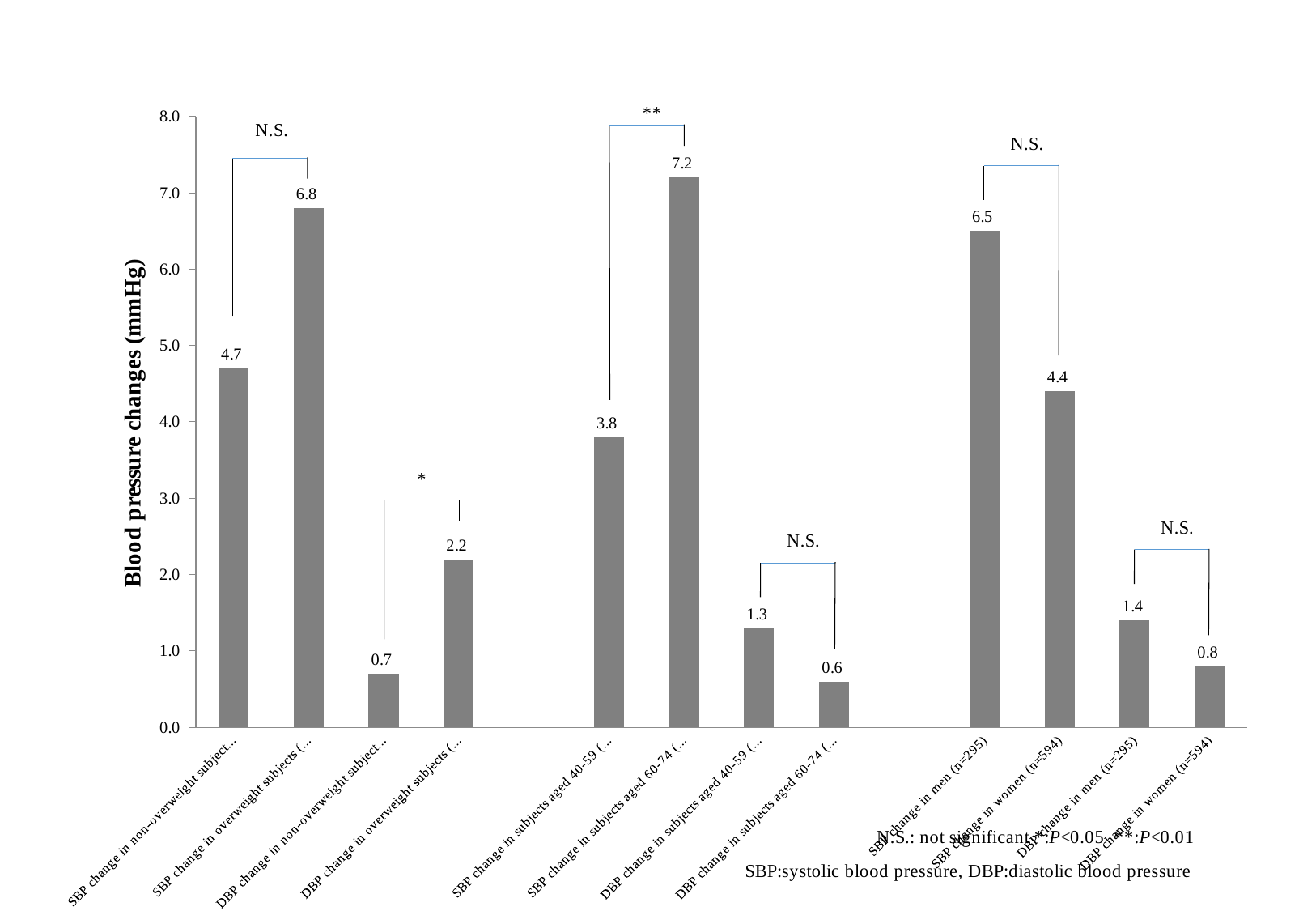

### Chart
| Category | |
|---|---|
| SBP change in non-overweight subjects (n=700) | 4.7 |
| SBP change in overweight subjects (n=189) | 6.8 |
| DBP change in non-overweight subjects (n=700) | 0.7 |
| DBP change in overweight subjects (n=189) | 2.2 |
| | None |
| SBP change in subjects aged 40-59 (n=533) | 3.8 |
| SBP change in subjects aged 60-74 (n=356) | 7.2 |
| DBP change in subjects aged 40-59 (n=533) | 1.3 |
| DBP change in subjects aged 60-74 (n=356) | 0.6 |
| | None |
| SBP change in men (n=295) | 6.5 |
| SBP change in women (n=594) | 4.4 |
| DBP change in men (n=295) | 1.4 |
| DBP change in women (n=594) | 0.8 |
